# When there is Confusion and Conflicts - Ask Delphi!

**Published:** 2015-07-01

**Authors:** Venkatachalam Raveenthiran, Yogesh Kumar Sarin

**Affiliations:** 1 Department of Pediatric Surgery Sri Ramasamy Memorial (SRM) Medical College SRM University, Kattankulathur, Chennai 603203, India; 2 Department of Pediatric Surgery Maulana Azad Medical College New Delhi, India

**Introduction:**

Controversy is an inseparable component of clinical medicine. Unlike in physical sciences, paradoxes and perplexities cannot be completely eliminated from biological sciences. The principal reason for this is the innumerable number of known and unknown co-variables that are sometimes difficult to control. Therefore modern clinicians bewildered by the contradictions often resort to several tactics that help them to overcome the “doctors’ dilemma”. These are broadly classified, in decreasing order of credibility, as Evidence Based Medicine (EBM), Clinical Guidelines and Consensus Statements. The innate growth potential of children, particularly that of newborn, adds the most difficult and dynamic dimension to the complexity of existing co-variables. No wonder that pediatric surgery remains a field full of contradictory opinions and disagreements. Surprisingly guidelines and consensus statements, which are very useful in resolving clinical disputes, are sparingly published in this specialty (1-5) and even the existing ones are of very poor quality.(6) This article is aimed to draw the attention of pediatric and neonatal surgeons to the usefulness of these conflict-solving tools.

**Role of Evidence-Based Medicine:**

EBM is useful when there is a large number of contradicting reports. It intends to objectively summarize the available evidences by applying judgment.(7, 8) Evidences are ranked according to their strength and reliability. Patient testimonials, case reports, and expert opinions without explicit critical appraisal are no longer accepted as evidence as they are liable for bias. Randomized controlled trials are preferred over uncontrolled studies and case series. When the published proofs are unsatisfactory or contradictory, techniques such as meta-analysis are used to generate higher quality evidences.(9,10) 

Although EBM has quickly become the "gold standard" in medicine, obviously it is not a panacea.(11-14) Clinical care is often dictated by personal factors such as quality of life and value systems. EBM cannot decide on these issues as they are not amenable for scientific methods and analysis. Although EBM may identify the best outcome of the available methods, it cannot settle the debate as to which can be considered the desirable outcome. The greatest setback of EBM is inaccessibility of all evidence either due to the problems of retrieval or due to publication bias (failure to publish negative results of trials). Further, effectiveness of treatment reported in clinical trials may be higher than that achieved in routine clinical practice because, close monitoring of patients during the study leads to much higher compliance rates. Critics of EBM would also observe that absence of evidence is not the evidence of absence. Data pooled from a number of studies make it more difficult to compare and control the co-variables than the data obtained from a defined set of patients in a given setting. In other words, EBM is applicable to populations, but not necessarily to individuals. RCTs, which are expensive, are biased because funding agencies according to their whims and fancies decide as to what gets investigated. Also, the quality of studies performed varies, making generalization of the results difficult. Methodological modifications intended to improve generalization will reduce the likelihood of detecting real differences between groups for a given sample size. Furthermore, EBM is frequently concerned with intermediate endpoints or clinical endpoints that may not bear any relevance to the ultimate outcome. For example, EBM may conclude Kasai portoenterostomy as a useful operation for biliary atresia by looking into the evidences of falling bilirubin levels (intermediate endpoint) or jaundice free survival (clinical endpoint); but the morbidity and mortality of late-onset portal hypertension (ultimate outcome) is completely sidelined in the analysis. From the foregoing it is obvious that EBM cannot always solve a problem. When EBM fails, the next sanctuary of bewildered clinicians is guidelines.

**Role of Clinical Guidelines:**

A “clinical guideline” (synonyms: medical guideline, clinical protocol or clinical practice guideline) aims to guide diagnostic and therapeutic decision-making in specific areas of healthcare. It is defined by authoritative examination of current, albeit inconclusive, evidences.(15,16) Algorithm of vomiting evaluation in newborn, diagnostic approach to disorders of sex development and therapeutic decision-making in necrotizing enterocolitis are examples of subjects suitable for clinical guidelines. They are usually produced at national or international levels by an independent panel of experts under the direction of medical associations or governmental bodies. Local doctors and hospital administrators may adapt and adopt them. Special computer software packages called “guideline execution engine” are used to generate, disseminate and implement clinical guidelines. (17) 

Use of guidelines raises the quality of health care and standardizes it by reducing many preventable errors.(18) For example, a checklist provided to nurses and junior doctors will timely remind them of interventions that might have otherwise been overlooked. It also improves cost effectiveness by eliminating unnecessary and non-specific procedures.(19) Guidelines also identify all possible decision options and their outcomes; integrates and groups them according to the practitioner’s level of experience; and thus renders decision-making easier. 

Skeptics, however, say that even some of the simple guidelines are not routinely followed in clinical practice.(20) The apprehension that standardized regulatory guidelines may impede scientific progress cannot be brushed aside (21,22) Guidelines are more suitable to resolve issues of competing priority. For example, they are useful in prioritizing the surgical approach of a neonate with coexisting tracheo-esophageal fistula (TEF), duodenal atresia and anorectal agenesis. But it must be realized that guidelines cannot resolve conflicts of technical concerns.(23,24) For example, guidelines cannot be provided as to which of the conflicting sets - transpleural versus extrapleural approach, ligation versus no ligation of azygos vein, intercostal drain versus no drain - is to be followed in TEF repair. Such technical disagreements demand development of consensus. Thus clinical guidelines when impossible or inapplicable, lead to the development of consensus statements.

**Role of Consensus Statements:**

Consensus statements, contrary to clinical guidelines, synthesize new information from recent or ongoing medical research blending it with a bit of intellectual prophecy. They do not give specific algorithms or guidelines for practice.(25,26) Consensus statements facilitate policy decisions on the basis of affordable cost, available expertise, technological access, political correctness and socioeconomic circumstances of the given clinical setting. Table 1 summarizes the differences between consensus statements and clinical guidelines. When valid evidences are lacking, consensus statements can be considered as a prelude to clinical guidelines and EBM. 

**Figure:**
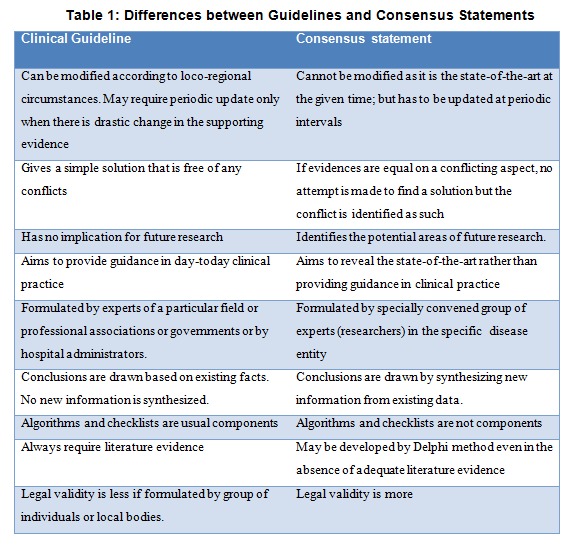
Table 1: Differences between Guidelines and Consensus Statements

**Nominal Group Technique:**

Medical consensus can be developed by two methods namely Nominal Group Technique (NGT - also known as expert panel) and Delphi process.(26) In NGT a set of selected experts - usually less than 10 in number - critically examine the available scientific data and concur as to the best of the alternatives. Each expert will contribute his/her views and the facilitator of the meeting will group the suggestions and call for deliberations on each of them. Finally the participants vote and rank the various ideas. The facilitator draws up the final list of rank order. The idea with overall high rank-vote is concluded as the common view point. The main objective of NGT is to counsel physicians as to the best of available options. NGT focuses on a single goal rather than qualitative examination of the group process per se. Selection of participants is likely to be biased and the question as to who can be considered as expert is contentious. Arriving at a consensus need not represent the correct answer; rather it may reflect the collective ignorance of the “experts”. The major drawback of NGT is the absence of opportunity for the participants to anonymously correct their misconception thereby help improving the efficiency of the process. There may still be some members left in the group who descent the high ranked idea. Thus the consensus of nominal group cannot really be called as consensus. This disadvantage is eliminated in Delhi technique. 

**The Delphi Technique:**

In circumstances where scientific data are completely or partially lacking, clinical guidelines as well as consensus statement of expert group becomes impractical. For example, there are hardly any scientific data on the usefulness of prophylactic antibiotics in antenatally diagnosed hydronephrosis. This is the occasion to use Delphi method to arrive at a consensus. This method was first developed in 1950s by RAND Corporation in forecasting the impact of technology on warfare. (26) It was subsequently adopted extensively in many business firms but has been used only occasionally in health care issues. Consensus arrived by this method is also considered to have legal validity. (27)

The Delphi method is a systematic interactive forecasting method based on independent inputs of selected experts. Details of this method are described elsewhere. (25,26) A person coordinating the Delphi method, known as a facilitator or panel director, facilitates the responses of the panel of experts, who are selected by virtue of their knowledge on a particular issue. The facilitator sends out questionnaires to the panel of experts. Responses of the panelists are collected, analyzed and the facilitator identifies common and conflicting viewpoints. Each round of questioning is followed by the feedback replies, usually presented anonymously. Thus, the experts are encouraged to revise their earlier answers in light of the replies of other members of the group. It is believed that the diversity of answers will progressively decrease with each round and the group will converge towards a unanimous solution. After several rounds, the process is complete. The median scores, rather than high score, determine the final answers. So Delphi method is a long-drawn tedious process. It gradually works towards synthesis, and building consensus in areas where there is lot of controversies. With the advent of long-distance internet communications, physical meeting of the members is no longer required. Recently, a consensus on risk assessment of necrotizing enterocolitis developed by e-Delphi method was reported. (29)

The following key characteristics of the Delphi method help the participants to focus on the issues at hand and separate Delphi from other methodologies:

(1) Structuring of information flow.

The initial contributions from the experts are collected in the form of answers to questionnaires and their comments to these answers. The facilitator controls the interactions among the participants by processing the information and filtering out irrelevant content. This avoids the negative effects of face-to-face panel discussions and solves the usual problems of group dynamics.

(2) Regular feedback.

Participants comment on their own forecasts, the responses of others and on the progress of the panel as a whole. At any moment they can revise their earlier statements. In regular group meetings participants tend to stick to previously stated opinions and often conform too much to group leader. This disadvantage is eliminated in Delphi method.

(3) Anonymity of participants.

Usually all participants maintain anonymity. Their identity is not revealed even after the completion of the final report. This stops the members from dominating over others by using their authority or personality. It also frees them to some extent from their personal biases, minimizes the "bandwagon effect" or "halo effect", allows them to freely express their opinions, and encourages open critique and admitting errors by revising earlier judgments.

The name "Delphi" derives from the Oracle of Delphi in ancient Greek mythology. The oracle at Delphi is of great authority and notorious ambiguity, where it was believed the god Apollo spoke through a priestess. Even the overall the track record of the Delphi method is mixed. (28) There have been many cases wherein the method produced poor results. However, some authors attribute this poor yield to faulty application of the method rather than to the inherent weakness of the method itself. It must also be realized that when forecasting is applied to science and technology, the degree of uncertainty is so great that accurate and correct predictions are not always feasible. It must also be appreciated that future developments do not depend on the predictions of the selected participant, but instead by unconventional thinker outside the group. One of the initial problems of the Delphi method was its inability to make complex forecasts with multiple factors. Potential future outcomes were usually considered as if they had no effect on each other. Later on, several extensions to the Delphi technique were developed to address this problem, such as cross-impact analysis.(30) Still, Delphi method is popularly used in forecasting single scalar indicators. It is to be remembered that this technique is vulnerable for abuse that it may give an appearance of community input when in reality the facilitator is directing the flow to a predetermined goal. Notwithstanding these demerits, Delphi technique can be considered analogous to a life buoy of a desperate sinking person. 

**Conclusion**

EBM is invoked when the clinical dispute is due to information overload. On the other hand, clinical guidelines are called for when the available evidences are inadequate and consensus statements when they are utterly contradictory or non-existent. If controversy is an inseparable component of Medicine, consensus statements and guidelines do not intend to remove it; but they only aim to subdue the conflicts until the truth is finally established by future research. Pediatric and neonatal surgeons should undertake high quality scientific research to solve controversies; until then consensus statements and guidelines should be developed to facilitate standardization of patient care.

## Footnotes

**Source of Support:** Nil

**Conflict of Interest:** Both the authors are editors of the journal. This article is not externally peer reviewed.

